# Supplementary material for: Structure of a VirD4 coupling protein bound to a VirB type IV secretion machinery
Source: EMBO J. 2017 Sep 18;36(20):3080–95. doi: 10.15252/embj.201796629 (PMC5916273; doi:10.15252/embj.201796629)
Supplement: Supplementary file 1 — Appendix [file EMBJ-36-3080-s001.pdf]

Appendix to

Structure of a VirD4 coupling protein bound to a VirB type IV secretion machinery

Adam Redzej*[a], Marta Ukleja*[a], Sarah Connery*[a], Martina Trokter[a], Catarina Felisberto-Rodrigues[a], Adam Cryar[b], Konstantinos Thalassinos[a,b], Richard D. Hayward[a,b], Elena V. Orlova[a] and Gabriel Waksman[a,b]

[a] Institute of Structural and Molecular Biology, Department of Biological Sciences, Birkbeck, London WC1E 7HX, United Kingdom
[b] Institute of Structural and Molecular Biology, Division of Biosciences, University College of London, London WC1E 6BT, United Kingdom

* These authors contributed equally to this work

To whom the correspondence should be addressed; email: g.waksman@mail.cryst.bbk.ac.uk
e.orlova@mail.cryst.bbk.ac.uk

Page	Description
S1	Appendix Cover Page
S2-S10	Appendix Table S1
S11	Appendix Table S2
S12	Appendix Table S3
S13-28	Appendix Table S4

APPENDIX TABLE S1 Plasmids used in this study

Entry	Plasmid	Description	Source
1	pMAK3	Wild-type conjugative plasmid, a variant of R388 with the T4SS DNA sequences being 97.3% identical.	Bradley & Cohen (1976)<br>Revilla et al. (2008)
2	pCDF_*trwABC*	pCDFDuet-1 vector containing the genes *trwA*, *trwB* and *trwC.*	This study
3	pCDF_*trwAC*	pCDFDuet-1 vector containing the genes *trwA* and *trwC.*	This study
4	pRSF_*oriT*	pRSFDuet-1 vector containing the *oriT* sequence from pMAK3 plasmid.	This study
5	pBADM11_$trwN_{/virB1}$-$trwE_{/virB10Strep}$	pBADM11 vector containing the genes $trwN_{/virB1}$, $trwL_{/virB2}$, $trwM_{/virB3}$, $trwK_{/virB4}$,	Low et al. (2014)

		trwJ$_{/virB5}$, *trwI*$_{/virB6}$, *trwH*$_{/virB7}$, *trwG*$_{/virB8}$, *trwF*$_{/virB9}$ and *trwE*$_{/virB10}$ from pMAK3 with a C-terminal Strep tag fusion to TrwE$_{/VirB10}$.	
6	pBADM11_*trwN*$_{/virB1}$-*trwD*$_{/virB11}$	pBADM11 vector expressing whole *trwN*$_{/virB1}$-*trwD*$_{/virB11}$ operon from pMAK3.	This study
7	pBADM11_*trwN*$_{/virB1}$-*trwE*$_{/virB10Strep}$_*trwD*$_{/virB11}$	pBADM11 vector expressing TrwN$_{/VirB1}$-TrwD$_{/VirB11}$ proteins from pMAK3 with TrwE$_{/VirB10}$ being expressed with a C terminal Strep tag. Ribosomal binding sites for *trwD*$_{/virB11}$ have been included within the construct.	This study
8	pBADM11_*trwN*$_{/virB1}$-*trwE*$_{/virB10Strep}$_*trwD*$_{/virB11\_His}$*trwB*$_{/virD4}$	pBADM11 vector expressing TrwN$_{/VirB1}$-TrwD$_{/VirB11}$ and TrwB$_{/VirD4}$ proteins from pMAK3 with TrwE$_{/VirB10}$ being expressed	This study

		with a C terminal Strep tag and $TrwB_{/VirD4}$ an N terminally located 10 Histidines. Ribosomal binding sites for both $trwD_{/virB11}$ and $trwB_{/virD4}$ genes have been included within the construct. Genes encoding $TrwD_{/VirB11}$ and $TrwB_{/VirD4}$ proteins have been included behind the $trwN_{/virB1}$-$trwE_{/virB10}$ cluster of genes.	
9	$\Delta trwL_{/virB2}$	Identical pBADM11_$trwN_{/virB1}$-$trwE_{/virB10Strep}$_$trwD_{/virB11_His}trwB_{/virD4}$ but with $trwL_{/virB2}$ gene deleted.	This study
10	$\Delta trwM_{/virB3}$	Identical pBADM11_$trwN_{/virB1}$-$trwE_{/virB10Strep}$_$trwD_{/virB11_His}trwB_{/virD4}$ but with $trwM_{/virB3}$ gene deleted.	This study
11	$\Delta trwK_{/virB4}$	Identical pBADM11_$trwN_{/virB1}$-	This study

		trwE$_{/virB10Strep}$ _*trwD*$_{/virB11\_His}$*trwB*$_{/virB4}$ but with *trwK*$_{/virB4}$ gene deleted.	
12	*ΔtrwJ*$_{/virB5}$	Identical pBADM11_*trwN*$_{/virB1}$-*trwE*$_{/virB10Strep}$ _*trwD*$_{/virB11\_His}$*trwB*$_{/virD4}$ but with *trwJ*$_{/virB5}$ gene deleted.	This study
13	*ΔtrwI*$_{/virB6}$	Identical pBADM11_*trwN*$_{/virB1}$-*trwE*$_{/virB10Strep}$ _*trwD*$_{/virB11\_His}$*trwB*$_{/virD4}$ but with *trwI*$_{/virB6}$ gene deleted.	This study
14	*ΔtrwG*$_{/virB8}$	Identical pBADM11_*trwN*$_{/virB1}$-*trwE*$_{/virB10Strep}$ _*trwD*$_{/virB11\_His}$*trwB*$_{/virD4}$ but with *trwG*$_{/virB8}$ gene deleted.	This study
15	*ΔtrwD*$_{/virB11}$	Identical pBADM11_*trwN*$_{/virB1}$-*trwE*$_{/virB10Strep}$ _*trwD*$_{/virB11\_His}$*trwB*$_{/virD4}$ but with *trwD*$_{/virB11}$ gene deleted.	This study
16	pBADM11_*trwB*$_{/virD4}$_*trwN*$_{/virB1}$-	Similar expression plasmid to	This study

	$trwE_{/virB10Strep}$_$trwD_{/virB11}$	pBADM11_$trwN_{/virB1}$-$trwE_{/virB10Strep}$_$trwD_{/virB11_His}trwB_{/virD4}$ with the main difference being the gene encoding for $TrwB_{/VirD4}$ is localized in front of the $trwN_{/virB1}$-$trwE_{/virB10}$ cluster of genes. Therefore additional ribosomal binding site for $trwN_{/virB1}$ gene has been included after the $trwB_{/virD4}$ gene.	
17	pBADM11_$trwB_{/virD4His}$_$trwN_{/virB1}$-$trwE_{/virB10Strep}$_$trwD_{/virB11}$	Identical to pBADM11_$trwB_{/virD4}$_$trwN_{/virB1}$-$trwE_{/virB10Strep}$_$trwD_{/virB11}$ but with a 10 histidines fused to the C terminus of $TrwB_{/VirD4}$.	This study
18	pBADM11_$trwB_{/virD4His}$_$trwN_{/virB1}$-$trwE_{/virB10Strep}$	Identical to pBADM11_$trwB_{/virD4His}$_$trwN_{/virB1}$-$trwE_{/virB10Strep}$_$trwD_{/virB11}$ but $trwD_{/virB11}$	This study

		gene deleted.	
19	pBADM11_*trwN*$_{/virB1}$-*trwE*$_{/virB10Strep\_His}$*trwB*$_{/virD4}$(*TrwB*$_{/VirD4236FLAG}$)	Identical to *ΔtrwD*$_{/virB11}$ but with a FLAG tag inserted in TrwB$_{/VirD4}$ in the place of the Thr 236.	This study
20	*ΔtrwM*$_{/virB3}$_*trwK*$_{/virB4FLAG}$	Identical to *ΔtrwM*$_{/virB3}$ but with a C terminal FLAG tag on TrwK$_{/VirB4}$.	This study
21	*ΔtrwM*$_{/virB3}$_*trwJ*$_{/virB5FLAG}$	Identical to *ΔtrwM*$_{/virB3}$ but with a C terminal FLAG tag on TrwJ$_{/VirB5}$.	This study
22	*ΔtrwM*$_{/virB3}$_*trwI*$_{/virB6FLAG}$	Identical to *ΔtrwM*$_{/virB3}$ but with a C terminal FLAG tag on TrwI$_{/VirB6}$.	This study
23	*ΔtrwM*$_{/virB3\_FLAG}$*trwG*$_{/virB8}$	Identical to *ΔtrwM*$_{/virB3}$ but with an N terminal FLAG tag on TrwG$_{/VirB8}$.	This study
24	*ΔtrwK*$_{/virB4\_FLAG}$*trwM*$_{/virB3}$	Identical to *ΔtrwK*$_{/virB4}$ but with an N terminal FLAG tag on TrwM$_{/VirB3}$.	This study

25	$\Delta trwK_{/virB4\_}trwJ_{/virB5FLAG}$	Identical to $\Delta trwK_{/virB4}$ but with a C terminal FLAG tag on $TrwJ_{/VirB5}$.	This study
26	$\Delta trwK_{/virB4\_}trwI_{/virB6FLAG}$	Identical to $\Delta trwK_{/virB4}$ but with a C terminal FLAG tag on $TrwI_{/VirB6}$.	This study
27	$\Delta trwK_{/virB4\_FLAG}trwG_{/virB8}$	Identical to $\Delta trwK_{/virB4}$ but with an N terminal FLAG tag on $TrwG_{/VirB8}$.	This study
28	$\Delta trwI_{/virB6\_}trwJ_{/virB5FLAG}$	Identical to $\Delta trwI_{/virB6}$ but with a C terminal FLAG tag on $TrwJ_{/VirB5}$.	This study
29	$\Delta trwG_{/virB8\_FLAG}trwM_{/virB3}$	Identical to $\Delta trwG_{/virB8}$ but with an N terminal FLAG tag on $TrwM_{/VirB3}$.	This study
30	$\Delta trwG_{/virB8\_}trwK_{/virB4FLAG}$	Identical to $\Delta trwG_{/virB8}$ but with a C terminal FLAG tag on $TrwK_{/VirB4}$.	This study
31	$\Delta trwG_{/virB8\_}trwJ_{/virB5FLAG}$	Identical to $\Delta trwG_{/virB8}$ but with a C terminal FLAG tag on $TrwJ_{/VirB5}$.	This study

32	*Δ*$trwG_{/virB8}$_$trwI_{/virB6FLAG}$	Identical to *Δ*$trwG_{/virB8}$ but with a C terminal FLAG tag on $TrwI_{/VirB6}$.	This study
33	pBADM11_$trwN_{/virB1}$-$trwE_{/virB10Strep}$ _$trwD_{/virB11_His}$$trwB_{/virD4}$($_{FLAG}$$trwM_{/virB3}$)	Identical to pBADM11_$trwN_{/virB1}$-$trwE_{/virB10Strep}$ _$trwD_{/virB11_His}$$trwB_{/virD4}$ but with an N terminal FLAG tag on $TrwM_{/VirB3}$.	This study
34	pBADM11_$trwN_{/virB1}$-$trwE_{/virB10Strep}$ _$trwD_{/virB11_His}$$trwB_{/virD4}$($trwK_{/virB4FLAG}$)	Identical to pBADM11_$trwN_{/virB1}$-$trwE_{/virB10Strep}$ _$trwD_{/virB11_His}$$trwB_{/virD4}$ but with a C terminal FLAG tag on $TrwK_{/VirB4}$.	This study
35	pBADM11_$trwN_{/virB1}$-$trwE_{/virB10Strep}$ _$trwD_{/virB11_His}$$trwB_{/virD4}$($trwJ_{/virB5FLAG}$)	Identical to pBADM11_$trwN_{/virB1}$-$trwE_{/virB10Strep}$ _$trwD_{/virB11_His}$$trwB_{/virD4}$ but with a C terminal FLAG tag on $TrwJ_{/VirB5}$.	This study
36	pBADM11_$trwN_{/virB1}$-$trwE_{/virB10Strep}$ _$trwD_{/virB11_His}$$trwB_{/virD4}$($trwI_{/virB6FLAG}$)	Identical to pBADM11_$trwN_{/virB1}$-$trwE_{/virB10Strep}$ _$trwD_{/virB11_His}$$trwB_{/virD4}$ but with a C terminal FLAG tag on $TrwI_{/VirB6}$.	This study

37	pBADM11_*trwN*$_{/virB1}$-*trwE*$_{/virB10Strep}$ _*trwD*$_{/virB11\_His}$*trwB*$_{/virD4}$(*$_{FLAG}$trwG*$_{/virB8}$)	Identical to pBADM11_*trwN*$_{/virB1}$-*trwE*$_{/virB10Strep}$ _*trwD*$_{/virB11\_His}$*trwB*$_{/virD4}$ but with an N terminal FLAG tag on TrwG$_{/VirB8}$.	This study
38	pBADM11_*$_{His_TEV}$trwB*$_{/virD4\Delta N70}$	Plasmid expressing cytoplasmic part of the TrwB$_{/VirD4}$ protein with an N terminal 6 histidines and TEV cleavage site.	This study
39	pCDFDuet-1	Cloning vector providing Spec resistance. Protein production is under control of the T7 promoter	Novagen
40	pRSFDuet-1	Cloning vector providing Kan resistance. Protein production is under control of the T7 promoter	Novagen

Appendix Table S2. Crosslinks identified within the $T4SS_{3\text{-}10\text{+}D4}$ sample where $TrwB_{/VirD4}$ is involved

Id	Crosslink	Error (ppm)	deltaS	Id-Score	FDR
AKQVTVAGVPMPR-VPLEIKQFANR-a2-b6	$HisTrwB_{/VirD4}$ (108) – $HisTrwB_{/VirD4}$ (493)	-1.8	0.28	53.06	0
DKDIILNPYDQR-TKGWSFFNEIR-a2-b2	$HisTrwB_{/VirD4}$ (171) – $HisTrwB_{/VirD4}$ (183)	2.5	0	49.34	0
FVLSDKLPEHVTMPDGDFSIR-GKTDEAEEWASYGR-a6-b2	$HisTrwB_{/VirD4}$ (287) – $HisTrwB_{/VirD4}$ (209)	1.7	0.64	33.05	0
VVAGLQSTSQLDDVYGVKEAQTLR-NTGKHHSTGR-a18-b4	$HisTrwB_{/VirD4}$ (398) – $HisTrwB_{/VirD4}$ (446)	2.5	0.39	30.14	0
NTGKHHSTGRALER-QESCIKALEDINR-a4-b6	$HisTrwB_{/VirD4}$ (446) – $TrwK_{/VirB4}$ (158)	0	0	27.9	0
KGDVIDAGAELER-IVSGGKLK-a1-b6	$TrwE_{/VirB10}$Strep (6) – $HisTrwB_{/VirD4}$ (98)	-6.3	0.35	32.61	0
KGDVIDAGAELER-VPLEIKQFANR-a1-b6	$TrwE_{/VirB10}$Strep (6) – $HisTrwB_{/VirD4}$ (493)	2.3	0.86	43.25	0

Appendix Table S3 Strains used in this study

Strain	Genotype	Source
DH5α	F^- *endA*1 *glnV44 thi-1 recA1 relA1 gyrA*96 *deoR nupG* Φ80d*lacZ*ΔM15 Δ(*lacZYA-argF*)U169, *hsdR*17(r_K^- m_K^+), $λ^–$	Thermo Fischer
TOP10	F- *mcrA* Δ(*mrr-hsd*RMS-*mcr*BC) Φ80*lac*ZΔM15 Δ *lac*X74 *rec*A1 *ara*D139 Δ(*araleu*)7697 *gal*U *gal*K *rps*L (StrR) *end*A1 *nup*G	Thermo Fischer
BL21Star™	F^- *ompT hsdSB* (r_B^- m_B^-) *gal dcm rne131* (DE3)	Thermo Fischer

Appendix Table S4 Primers used in this study

15bp overlapping sequences present on the primers are marked in small letters, restriction sites and additional sequences added to the genes are marked in bold.

Entry	Name of the primers	Sequence of the primers	Name of the construct cloned using the primers	Description
1	opRoriT_*Asc*I_FW and opRoriT_*Asc*I_Rev	TATA**GGCGCGCC**ACTCATTTTCTGCATCATTG and AATT**GGCGCGCC**ACCGCCTCGTCCTC	pRSF_*oriT*	These primers were used to generate a PCR fragment of R388 oriT region flanked on both the 5' and 3' end with an AscI restriction site
2	otrwABC_*Asc*I_FW and otrwABC_*Xho*I_Rev	CTA**GGCGCGCC**ATGCTAGGATTGAAC and GAT**CTCGAGTCA**TTACCTTCCGGCCTC	pCDF_*trwABC*	These primers were used to generate a PCR fragment with a *trwABC* genes in between *AscI* and *XhoI* sites (marked in bold)
3	Rbs_TrwC_FW and trwArbs_Rev	aggggactatctaatGCTCAGTCACATGG and attagatagtcccctTCAATCCTCCTTCCCCTCC	pCDF_*trwAC*	These primers were used to linearize pCDF_*trwABC* vector excluding the *trwB* gene
4	B1-11_FW and B1-11_Rev	caggaggaattaaccATGGCACTGGCAGAGTTCGCGG and agccaagctctcttaAGCCATCTTGGACTTGGAAAAGATCG	pBADM11_*trwN*$_{/virB1}$-*trwD*$_{/virB11}$	These primers were used to generate a PCR fragment, which included all the *trwN*$_{/virB1}$-*trwD*$_{/virB11}$ genes.

5	pBADM11_FW and pBADM11_Rev	taagagagcttggctGTTTTGGCGG and ggttaattcctcctgTTAGCCC	pBADM11_*trwN*$_{/virB1}$-*trwD*$_{/virB11}$	These primers were used to linearize the pBADM11 vector.
6	lin_pBAD_B1-B10_FW and lin_pBAD_B1-B10_Rev	gagagcttggctgttTTGGCGG and ttatttttcgaactgCGGGTGGCTCC	pBADM11_*trwN*$_{/virB1}$-*trwE*$_{/virB10Strep}$ _*trwD*$_{/virB11}$	These primers were used to linearize the pBADM11_*trwN*$_{/virB1}$-*trwE*$_{/virB10Strep}$ vector.
7	rbsB11_FW and B11_Rev	cagttcgaaaaataaAGGAAATAAATAATGTCTACAGTCTCG and aacagccaagctctcTTAAGCCATCTTGGACTTGG	pBADM11_*trwN*$_{/virB1}$-*trwE*$_{/virB10Strep}$ _*trwD*$_{/virB11}$	These primers were used to generate a PCR fragment containing the *trwD*$_{/virB11}$ gene and its ribosomal binding site.
8	pBAD_FW and B11_2_Rev	gagagcttggctgttTTGGCGGATGAGAG and ttaagccatcttggaCTTGGAAAAGATCGGG	pBADM11_*trwN/virB1*-*trwE*$_{/virB10Strep}$ _*trwD*$_{/virB11}$_*trwB*$_{/virD4}$	These primers were used to linearize the pBADM11_*trwN*$_{/virB1}$-*trwE*$_{/virB10Strep}$ _*trwD*$_{/virB11}$ vector.
9	B11_rbs_D4_FW and D4_BAD_Rev	tccaagatggcttaaGGAGGATTGAGATGCATCCAGACG and aacagccaagctctcTTAGATAGTCCCCTCAACAAAGG	pBADM11_*trwN*$_{/virB1}$-*trwE*$_{/virB10Strep}$ *trwD*$_{/virB11}$_*trwB*$_{/virD4}$	These primers were used to generate a PCR product of the *trwB*$_{/virD4}$ gene and its ribosomal binding site.
10	B4_FW and B11_rbs_Rev	actcgatgagaaataCCGGGAGAGCTTCACCGGCTATAACC and gtgatgatggtgatgCATCTCAATCCTCCTTAAGCCATCTTGGACTTGG	pBADM11_*trwN*$_{/virB1}$-*trwE*$_{/virB10Strep}$ *trwD*$_{/virB11\_His}$*trwB*$_{/virD4}$	These primers were used to generate a PCR fragment containing part of the *trwK*$_{/virB4}$ gene and genes upstream to it including *trwD*$_{/virB11}$ gene. As a template the

				pBADM11_*trwN*$_{/virB1}$-*trwE*$_{/virB10Strep}$ *trwD*$_{/virB11}$_*trwB*$_{/virD4}$ vector was used.
11	10His_D4_FW and B4_Rev	catcaccatcatcacCACCATCACCACCACCATCCAGACGATCAAAGAAAGG and tatttctcatcgagtTGACGGCAAAACACG	pBADM11_*trwN*$_{/virB1}$-*trwE*$_{/virB10Strep}$ *trwD*$_{/virB11\_His}$*trwB*$_{/virD4}$	These primers were used to generate a PCR fragment of *trwB*$_{/virD4}$ gene with a sequence coding for 10 histidines on the 5' end of the *trwB*$_{/virD4}$ gene and linearize the pBADM11 vector with *trwN*$_{/virB1}$-*trwK*$_{/virB4}$ genes included.
12	B3_FW and B11_9993_Rev	ccgggcgaaagctaaAGGGGGACAGTCATGAAGCC and gcgcttgtagagcacGCACTTTTCG	*ΔtrwL*$_{/virB2}$	These primers were used to generate PCR fragment which included *trwM*$_{/virB3}$ and upstream localized genes up to the middle part of *trwD*$_{/virB11}$ gene
13	B11_10003_FW and KorA_Rev	gtgctctacaagcgcAACATCATCATCGCG and ttagctttcgcccggAGGTAGTTCAGG	*ΔtrwL*$_{/virB2}$	These primers were used to generate PCR fragment which included the rest of the *trwD*$_{/virB11}$ gene and upstream localized genes as well as the backbone of the pBADM11 vector and the

				sequence of *trwN*$_{/virB1}$ gene
14	B4_FW and B11_9993_Rev	atgctgtttacctaaAGGAGATAGGCATGGGGGC and gcgcttgtagagcacGCACTTTTCG	*Δ**trwM*$_{/virB3}$	These primers were used to generate PCR fragment which included *trwK*$_{/virB4}$ and upstream localized genes up to the middle part of *trwD*$_{/virB11}$ gene
15	B11_10003_FW and B2_Rev	tgctctacaagcgcaACATCATCATCGCG and ttaggtaaacagcatGGCCGTAATCTGG	*Δ**trwM*$_{/virB3}$	These primers were used to generate PCR fragment which included the rest of the *trwD*$_{/virB11}$ gene and upstream localized genes as well as the backbone of the pBADM11 vector and the sequence of *trwL*$_{/virB2}$ gene
16	B5_FW and D4_11260_Rev	cgaaaaaggagatagAGGAGCGACGTATGAAGAAGC and gtccagtggaacaatTCGCGCATGG	*Δ**trwK*$_{/virB4}$	These primers were used to generate PCR fragment which included *trwJ*$_{/virB5}$ and upstream localized genes up to the middle part of *trwB*$_{/virD4}$ gene
17	D4_11246_FW and B3_Rev	attgttccactggacAACCATCGCCACG and ctatctcctttttcgGTAGTTAGCGGG	*Δ**trwK*$_{/virB4}$	These primers were used to generate PCR fragment which included

				the rest of the *trwB*$_{/virD4}$ gene and upstream localized genes as well as the backbone of the pBADM11 vector and the sequence of *trwM*$_{/virB3}$ gene
18	Eex_FW<br>and<br>AMP_Rev	aggagcgacgtatgaAGGAATGCCGACTATCCAATG<br>and<br>gctcttgcccggcgtCAACACG	*ΔtrwJ*$_{/virB5}$	These primers were used to generate PCR fragment which included *eex* and upstream localized genes up to the middle part of ampicillin resistance cassette
19	AMP_FW<br>and<br>B4_Rev	acgccgggcaagagcAACTCGGTCG<br>and<br>tcatacgtcgctcctTTCGGCTTTCACACGG	*ΔtrwJ*$_{/virB5}$	These primers were used to generate PCR fragment which included the rest of the ampicillin resistance cassette and upstream localized genes as well as the backbone of the pBADM11 vector and the sequence of *trwK*$_{/virB4}$ gene
20	B7_FW<br>and<br>AMP_Rev	aggagaggtgttgacGTGAAAACC<br>and<br>gctcttgcccggcgtCAACACG	*ΔtrwI*$_{/virB6}$	These primers were used to generate PCR fragment which included *trwH*$_{/virB7}$ and upstream localized genes up to the

				middle part of ampicillin resistance cassette
21	AMP_FW and eex_Rev	acgccgggcaagagcAACTCGGTCG and gtcaacacctctcctCTACCGGATTTTTGGCATCCCGTCG	*Δtrw* $I_{/virB6}$	These primers were used to generate PCR fragment which included the rest of the ampicillin resistance cassette and upstream localized genes as well as the backbone of the pBADM11 vector and the sequence of *eex* gene
22	B9_FW and ColE1_Rev	aggggggcaccatgaAGAAACTAGC and ccttatccggtaactATCGTCTTGAGTCC	*Δtrw* $G_{/virB8}$	These primers were used to generate PCR fragment which included *trw* $F_{/virB9}$ and upstream localized genes up to the middle of the ColE1 region
23	ColEI_FW and B7_Rev	agttaccggataaggCGCAGCGG and tcatggtgccccccctTCATAGTGCCCCTCGCTGAATTTCC	*Δtrw* $G_{/virB8}$	These primers were used to generate PCR fragment, which included the rest of the ColEI region and upstream localized genes as well as the backbone of the pBADM11 vector and the sequence of *trw* $H_{/virB7}$ gene.

24	rbsHis_D4_FW<br>and<br>B4_2356_Rev	cagttcgaaaaataaGGAGGATTGAGATGCATCACC<br>and<br>tatttctcatcgagtTGACGGCAAAACACGTTGTCAAACTCG	$\Delta trwD_{/virB11}$	These primers were used to generate PCR fragment, which included the $_{\mathrm{His}}trwB_{/virD4}$ gene, vector backbone and the genes from $trwN_{/virB1}$ to the part of the sequence of $trwK_{/virB4}$ gene.
25	B4_2342_2_FW<br>and<br>B10Strep_Rev	actcgatgagaaataCCGGGAGAGCTTCACCGGC<br>and<br>ttatttttcgaactgCGGGTGGCTCCACTTAGTGG	$\Delta trwD_{/virB11}$	These primers were used to generate PCR fragment, which included the rest of the $trwK_{/virB4}$ region and upstream localized genes up to $trwE_{/virB10\mathrm{Strep}}$ gene.
26	D4_rbs_B1_FW<br>and<br>pBAD_Rev	taaaggaggattgagATGGCACTGGCAGAGTTCGCG<br>and<br>ggttaattcctcctgTTAGCCCAAAAAACGGGTATGG	pBADM11_$trwB_{/virD4}$_$trwN_{/virB1}$-$trwE_{/virB10Strep}$ $trwD_{/virB11}$	These primers were used to linearize the pBADM11_$trwN_{/virB1}$-$trwE_{/virB10Strep}$ $trwD_{/virB11}$ vector.
27	pBAD_D4_FW<br>and<br>D4_rbs_Rev	caggaggaattaaccATGCATCCAGACGATCAAAGAAAGG<br>and<br>ctcaatcctcctttaGATAGTCCCCTCAACAAAGGCCGG	pBADM11_$trwB_{/virD4}$_$trwN_{/virB1}$-$trwE_{/virB10Strep}$ $trwD_{/virB11}$	These primers were used to generate a PCR product of the $trwB_{/virD4}$ gene.
28	R3_10His_rbs_B1_FW<br>and<br>B10_8916_Rev	atcatcaccaccatcACCACCACTAAAGGAGGATTGAGATGGCACTGG<br>and<br>atggcaggtagtcatGCCGGGTTGCGTCGTCAC	pBADM11_$trwB_{/virD4His}$_ $trwN_{/virB1}$-$trwE_{/virB10Strep}$ $trwD_{/virB11}$	These primers were used to generate PCR fragment, which included the $trwN_{/virB1}$ and genes

		AAGGC		located upstream including part of the *trwE*/virB10 gene.
29	B10_8902_FW and R3_D4_10His_Rev	atgactacctgccatCTGACGC and gatggtggtgatgatGGTGATGGATAGTCCCCTCAACAAAGGC	pBADM11_*trwB*/virD4His_ *trwN*/virB1-*trwE*/virB10Strep *trwD*/virB11	These primers were used to generate PCR fragment, which included the rest of the *trwE*/virB10Strep gene and upstream localized genes including the backbone of the vector and the *trwB*/virD4 gene with additional sequence encoding for polyhistidine tag at the 3' end of the *trwB*/virD4 gene.
30	deltaB11_FW and B4_2356_Rev	cagttcgaaaaataaTAAGAGAGCTTGGCTGTTTTGGCGG and tatttctcatcgagtTGACGGCAAAACACGTTGTCAAACTCG	pBADM11_*trwB*/virD4His_ *trwN*/virB1-*trwE*/virB10Strep	These primers were used to generate PCR fragment which included *trwB*/virD4, linearized backbone of the pBADM11 vector and the sequence of the genes starting from *trwN*/virB1 to the middle part of *trwK*/virB4 gene
31	B4_2342_FW and deltaB11_Rev	actcgatgagaaataCCGGGAGAGC and ttatttttcgaactgCGGGTGGCTCCACTTAGTG	pBADM11_*trwB*/virD4His_ *trwN*/virB1-*trwE*/virB10Strep	These primers were used to generate PCR fragment which included

		G		the rest of the *trwK/virB4* gene and upstream localized genes including *trwE/virB10Strep* gene
32	B4_loop_FW and D4loop_Flag_Rev	actcgatgagaaataCCGGGAGAGCTTCACCGG and tatcgtcgtcatcctTATAATCACCAATAAGCGCCAATTTTTTAGC	pBADM11_*trwN/virB1-trwE/virB10Strep _HistrwB/virD4(TrwB/VirD4236FLAG)*	These primers were used to generate PCR fragment, which included part of the *trwK/virB4* gene and upstream localized genes including part of the *trwB/virD4* gene with additional sequence encoding for FLAG tag at the position of the *trwB/virD4* gene encoding for the Thr236.
33	D4loop_Flag_FW and B4_loop_Rev	aggatgacgacgataAATTCCCTTCCATGCGCGAATTGTTCCACTG and tatttctcatcgagtTGACGGCAAAACACGTTGTCAAACTCGGC	pBADM11_*trwN/virB1-trwE/virB10Strep _HistrwB/virD4(TrwB/VirD4236FLAG)*	These primers were used to generate PCR fragment which included the rest of the *trwB/virD4* gene and the vector together with the *trwN/virB1-trwK/virB4* genes.
34	B10forB3_FW and B10forB3_Rev	tacctgccatctgacGCGGGACGTTTACTCGACCAG and atttatcgtcgtcatCCTTATAATCCATGACTGTCCCCCTTTAGGTAAAC	*ΔtrwX/virBY_FLAGtrwM/virB3 (X=K,G; Y=4,8)*	These primers were used to linearize plasmids *ΔtrwK/virB4 and ΔtrwG/virB8* and to incorporate a FLAG tag

				encoding sequence on the 5' end of the *trwM/virB3* gene. The generated PCR fragment included part of the *trwE/virB10* gene and genes upstream as well as the vector and the *trwN/virB1* to *trwM/virB3* genes with additional sequence encoding for FLAG tag at the 5' end of the *trwM/virB3* gene.
35	B3Flag_FW and B10forB3_Rev	atgacgacgataaatTCGGCAGCGGCAAGCCGCCACAGCAGCAGCACG and gtcagatggcaggtaGTCATGCCGGGTTGCGTCGTCACAAGGCGC	*ΔtrwX/virBY_FLAGtrwM/virB3 (X=K,G; Y=4,8)*	These primers were used to linearize plasmids *ΔtrwK/virB4 and ΔtrwG/virB8* and to incorporate a FLAG tag encoding sequence on the 5' end of the *trwM/virB3* gene. The generated PCR fragment included part of the *rest of the trwM/virB3* gene and genes upstream of it including part of the *trwE/virB10* gene.
36	B4Flag_FW and	attataaggatgacgACGATAAATTCTGAAAGGAGCGACGTATGAAGAAG	*ΔtrwX/virBY_trwK/virB4FLAG (X=M,G; Y=3,8)*	These primers were used to linearize plasmids

	D4forB4_Rev	and<br>ccgaagccctgccttTCGGCCTTTGGTGAGTGCG		*ΔtrwM$_{/virB3}$ and ΔtrwG$_{/virB8}$* and to incorporate a FLAG tag encoding sequence on the 3’ end of the *trwK$_{/virB4}$* gene. The generated PCR fragment included part of the *trwK$_{/virB4}$* gene and genes upstream including part of the *trwB$_{/virD4}$* gene with additional sequence encoding for FLAG tag at the 3’ end of the *trwK$_{/virB4}$* gene.
37	D4forB4_FW<br>and<br>B4Flag_Rev	aaggcagggcttcggGTTGTGGCGGGCCTGCAATCGACCTCG<br>and<br>cgtcatccttataatCGCCGCTGCCTACGTCGCTCCTTTCGGCTTTCAC	*ΔtrwX$_{/virBY}$_trwK$_{/virB4FLAG}$ (X=M,G; Y=3,8)*	These primers were used to linearize plasmids *ΔtrwM$_{/virB3}$ and ΔtrwG$_{/virB8}$* and to incorporate a FLAG tag encoding sequence on the 3’ end of the *trwK$_{/virB4}$* gene. The generated PCR fragment included the rest of the *trwB$_{/virD4}$* gene as well as the vector and the *trwN$_{/virB1}$* to *trwK$_{/virB4}$* genes with additional

				sequence encoding for FLAG tag at the 3' end of the *trwK*$_{/virB4}$ gene.
38	AmpforB5_FW and B5Flag_Rev	ccataccaaacgacgAGCGTGACACC and cgtcatccttataatCGCCGCTGCCTTGGATAG TCGGCATTCCTTGGTTTC	*ΔtrwX*$_{/virBY}$_*trwJ*$_{/virB5FLAG}$ *(X=M,K,I,G; Y=3,4,6,8)*	These primers were used to linearize plasmids *ΔtrwM*$_{/virB3}$, *ΔtrwK*$_{/virB4}$, *ΔtrwI*$_{/virB6}$ *and ΔtrwG*$_{/virB8}$ and to incorporate a FLAG tag encoding sequence on the 3' end of the *trwJ*$_{/virB5}$ gene. The generated PCR fragment included part of the Ampicillin resistance cassette and genes located upstream including the vector and the *trwN*$_{/virB1}$ to *trwJ*$_{/virB5}$ genes with additional sequence encoding for FLAG tag at the 3' end of the *trwJ*$_{/virB5}$ gene.
39	B5Flag_FW and AmpforB5_Rev	attataaggatgacgACGATAAATTCTGAAAG GAATGCCGACTATCCAATG and cgtcgtttggtatggCTTCATTCAG	*ΔtrwX*$_{/virBY}$_*trwJ*$_{/virB5FLAG}$ *(X=M,K,I,G; Y=3,4,6,8)*	These primers were used to linearize plasmids *ΔtrwM*$_{/virB3}$, *ΔtrwK*$_{/virB4}$, *ΔtrwI*$_{/virB6}$ *and ΔtrwG*$_{/virB8}$ and to incorporate a FLAG tag encoding sequence on the 3' end of

				the *trwJ*$_{/virB5}$ gene. The generated PCR fragment included the end of the *trwJ*$_{/virB5}$ gene with additional sequence encoding for FLAG tag at the 3’ end of the *trwJ*$_{/virB5}$ gene and genes upstream including the rest of the Ampicillin resistance cassette.
40	B6Flag_FW and ColE1forB6_Rev	ttataaggatgacgaCGATAAATTCTAGTTGTCAGGGTGTAAAGCGGCGG and catagctcacgctgtAGGTATCTCAGTTCGGTGTAGGTC	*ΔtrwX*$_{/virBY}$*_trwI*$_{/virB6FLAG}$ *(X=M,K,G; Y=3,4,8)*	These primers were used to linearize plasmids *ΔtrwM*$_{/virB3}$, *ΔtrwK*$_{/virB4}$ *and ΔtrwG*$_{/virB8}$ and to incorporate a FLAG tag encoding sequence on the 3’ end of the *trwI*$_{/virB6}$ gene. The generated PCR fragment included the end of the *trwI*$_{/virB6}$ gene with additional sequence encoding for FLAG tag at the 3’ end of the *trwI*$_{/virB6}$ gene and *ColE1* region of the vector.
41	ColE1forB6_FW and B6Flag_Rev	acagcgtgagctatgAGAAAGCGCCACG and tcgtcatccttataaTCGCCGCTGCCCCCTGCTT	*ΔtrwX*$_{/virBY}$*_trwI*$_{/virB6FLAG}$ *(X=M,K,G; Y=3,4,8)*	These primers were used to linearize plasmids *ΔtrwM*$_{/virB3}$, *ΔtrwK*$_{/virB4}$

		TGCGTCCCCGGAAGTAAC		*and ΔtrwG/virB8* and to incorporate a FLAG tag encoding sequence on the 3' end of the *trwI/virB6* gene. The generated PCR fragment included the rest of the ColE1 region of the vector and genes upstream including *trwI/virB6* gene with additional sequence encoding for FLAG tag at the 3' end of the *trwI/virB6* gene.
42	B1forB8_FW and B8Flag_Rev	gattctccaatggctATGTGCAGAAAGTGG and tcgtcgtcatccttaTAATCCATAGTGCCCCTC GCTTCATAGTGCCCCTCGCTG	*ΔtrwX/virBY_FLAGtrwG/virB8 (X=M,K; Y=3,4)*	These primers were used to linearize plasmids *ΔtrwM/virB3* and *ΔtrwK/virB4* and to incorporate a FLAG tag encoding sequence on the 5' end of the *trwG/virB8* gene. The generated PCR fragment included the *trwN/virB1*-*trwG/virB8* genes with a FLAG tag at the 5' beginning of the *trwG/virB8* sequence.
43	B8Flag_FW	taaggatgacgacgaTAAATTCAGCGGCAGCA	*ΔtrwX/virBY_FLAGtrwG/virB8*	These primers were used

	and B1forB8_Rev	AGAAGCAACCAAAACCGGTCAAG and agccattggagaatcCGCGCTGGAAATTG	*(X=M,K; Y=3,4)*	to linearize plasmids *ΔtrwM$_{/virB3}$* and *ΔtrwK$_{/virB4}$* and to incorporate a FLAG tag encoding sequence on the 5' end of the *trwG$_{/virB8}$* gene. The generated PCR fragment included the rest of the *trwG$_{/virB8}$* gene and genes located upstream including the vector sequence.
44	KorA_FW and B7_Rev	agtcgtgaagcgttcGGAAAAGGAATGG and gcacacgccgacaatAGCCCCG	pBADM11_*trwN$_{/virB1}$*-*trwE$_{/virB10Strep}$* _*trwD$_{/virB11_His}$trwB$_{/virD4}$*(*$_{FLAG}$trwX$_{/virBY}$ or trwX$_{/virBYFLAG}$*) *(X=M,K,J,I,G; Y=3,4,5,6,8)*	These primers were used to incorporate FLAG tag sequence at 5' end of the *trwM$_{/virB3}$*, *trwG$_{/virB8}$* or 3' end of the *trwK$_{/virB4}$*, *trwJ$_{/virB5}$*, *trwI$_{/virB6}$* in the pBADM11_*trwN$_{/virB1}$*-*trwE$_{/virB10Strep}$* _*trwD$_{/virB11_His}$trwB$_{/virD4}$* construct.
45	B7_FW and KorA_Rev	attgtcggcgtgtgcATCGGCTCCCAAG and gaacgcttcacgactGTTACTTCGCGCACAG	pBADM11_*trwN$_{/virB1}$*-*trwE$_{/virB10Strep}$* _*trwD$_{/virB11_His}$trwB$_{/virD4}$*(*$_{FLAG}$trwX$_{/virBY}$ or trwX$_{/virBYFLAG}$*) *(X=M,K,J,I,G; Y=3,4,5,6,8)*	These primers were used to incorporate FLAG tag sequence at 5' end of the *trwM$_{/virB3}$*, *trwG$_{/virB8}$* or 3' end of the *trwK$_{/virB4}$*, *trwJ$_{/virB5}$*, *trwI$_{/virB6}$* in the

				pBADM11_*trwN*$_{/virB1}$-*trwE*$_{/virB10Strep}$_*trwD*$_{/virB11\_His}$*trwB*$_{/virD4}$ construct.
46	HTTrwB_FW and HTTrwBΔ_Rev	ctctacttccaatcgTTGAATAGCGTCGGACAAGGC and aacagccaagctctcTTAGATAGTCCCCTCAACAAAGG	pBADM11$_{_His_TEV}$*trwB*$_{/virD4\Delta N70}$	These primers were used to generate a PCR fragment, which includes the sequence of the *trwB*$_{/virD4}$ without the codons encoding for the first 70 residues.
47	pBAD6HTEV_FW and pBAD6HTEV_Rev	gagagcttggctgttTTGGCGG and cgattggaagtagag**GTTCTCGTGGTGATGATGGTGATGCATGGTTAATTCCTCCTGTTAGC**	pBADM11$_{_His_TEV}$ *trwB*$_{/virD4\Delta N70}$	These primers were used to linearize the pBADM11 and add the sequence encoding for TEV cleavage site and 6 histidines after the promoter region (in bold).
