# Supplementary material for: Structure of a VirD4 coupling protein bound to a VirB type IV secretion machinery
Source: EMBO J. 2017 Sep 18;36(20):3080–95. doi: 10.15252/embj.201796629 (PMC5916273; doi:10.15252/embj.201796629)
Supplement: Supplementary file 2 — Expanded View Figures PDF [file EMBJ-36-3080-s002.pdf]

Expanded View Figures

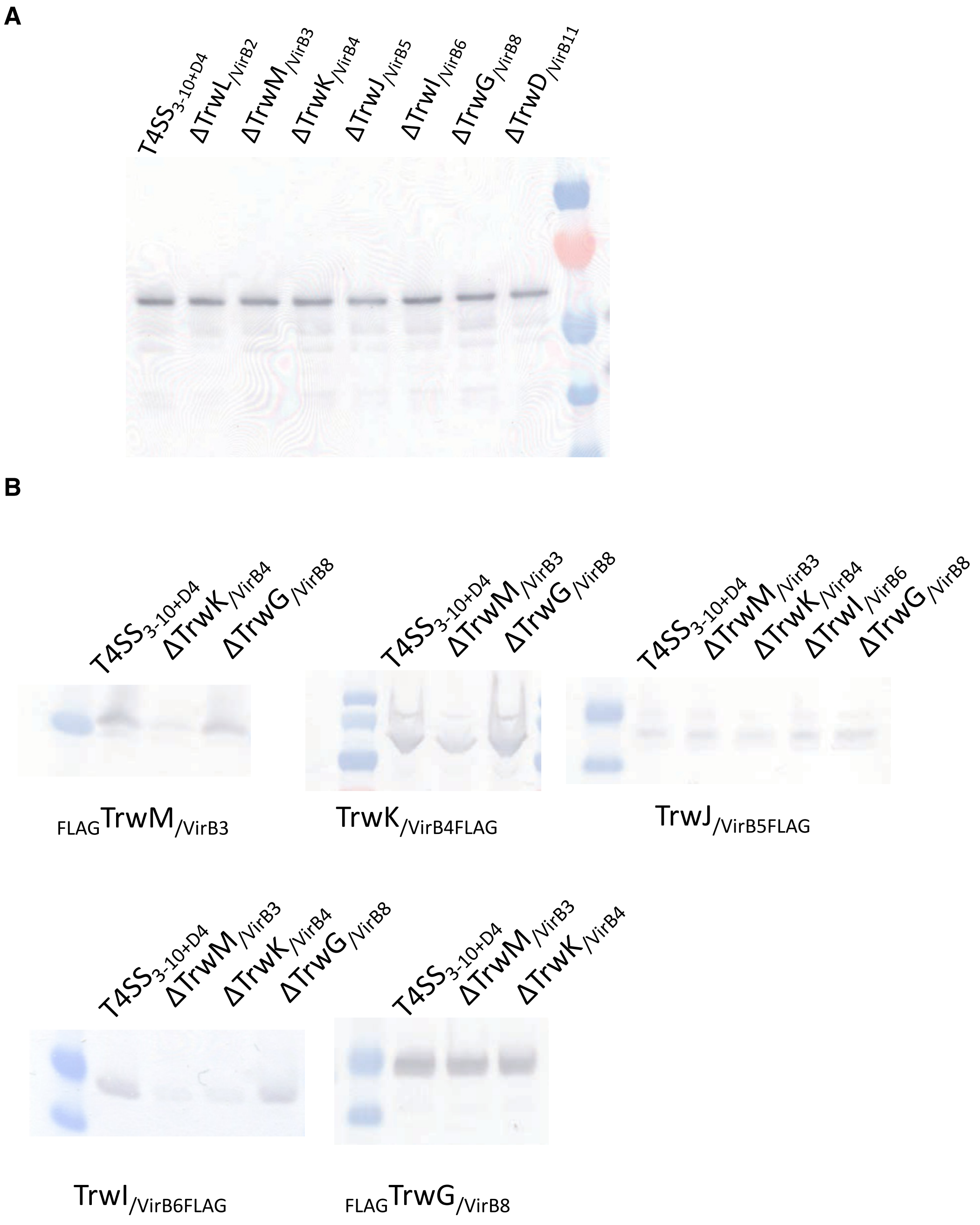

Figure EV1. Western blot analysis aimed at ascertaining that all proteins are expressed.

A Western blot visualizing the expression levels of $TrwB_{/VirD4}$ within different deletion constructs demonstrating that there is no polar effect observed for the expression of $TrwB_{/VirD4}$ due to gene deletion and that similar quantities of materials were loaded on the gel.

B Western blot visualizing expression of components which are lost during purification of the $T4SS_{3\text{-}10+D4}$ complex upon deletion of single T4S secretion components. The construct used for expression is shown at the top of each lane while the FLAG-tagged protein detected by anti-FLAG tag antibodies is shown under the panel.

Source data are available online for this figure.

© 2017 The Authors

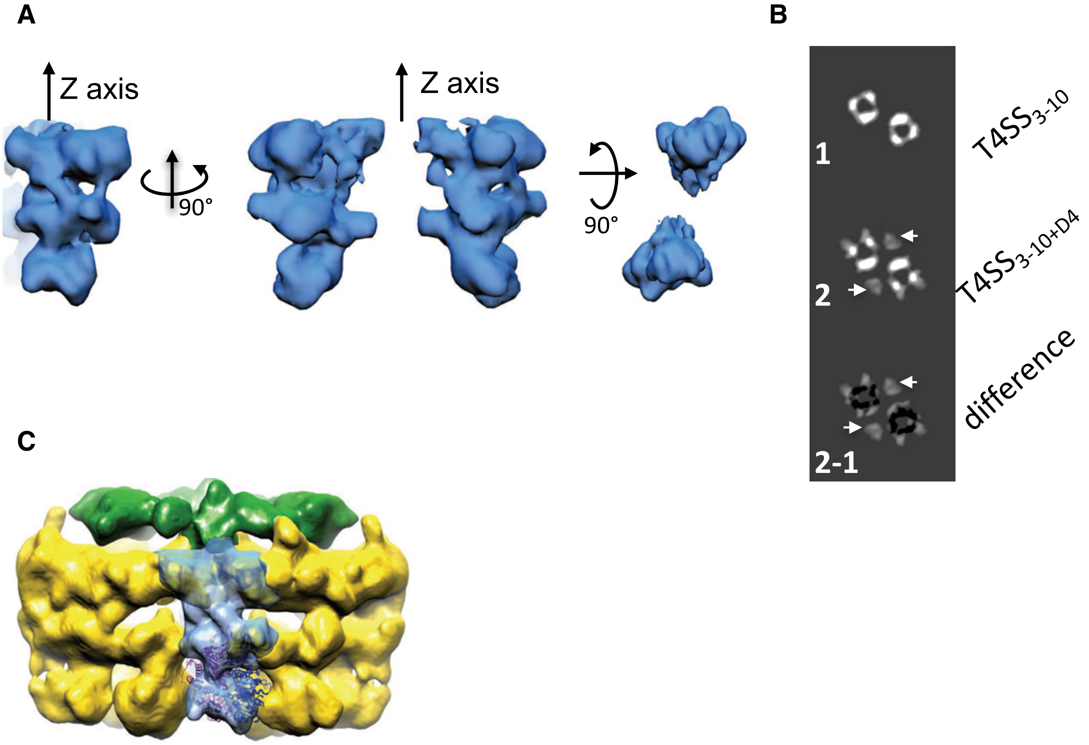

Figure EV2. Difference map, comparison of cross sections of the bottom part of the IMC of the $T4SS_{3\text{-}10\text{+}D4}$ and the $T4SS_{3\text{-}10}$ structures, fitting of the $TrwB_{/VirD4\Delta N70}$ X-ray structures into the map of the IMC of the $T4SS_{3\text{-}10\text{+}D4}$.

A Difference map between the IMC $T4SS_{3\text{-}10\text{+}D4}$ and the $T4SS_{3\text{-}10}$ structures corresponding to $TrwB_{/VirD4}$ densities. Left: front view. Middle panel: side view (the structure is rotated on 90 degrees along a vertical axis). Right panel: bottom view.

B Comparison of the cross sections of the bottom part of the IMC of the $T4SS_{3\text{-}10}$ (top), the $T4SS_{3\text{-}10\text{+}D4}$ (middle) and the difference between them (bottom). Extra density corresponding to $TrwB_{/VirD4}$ is indicated by white arrows.

C Fitting of two $TrwB_{/VirD4}$ cytoplasmic domain structures into the blue density of Fig 4B illustrating that this density can accommodate only two of these molecules, not six. One subunit is shown in magenta and another one in dark blue.

 © 2017 The Authors

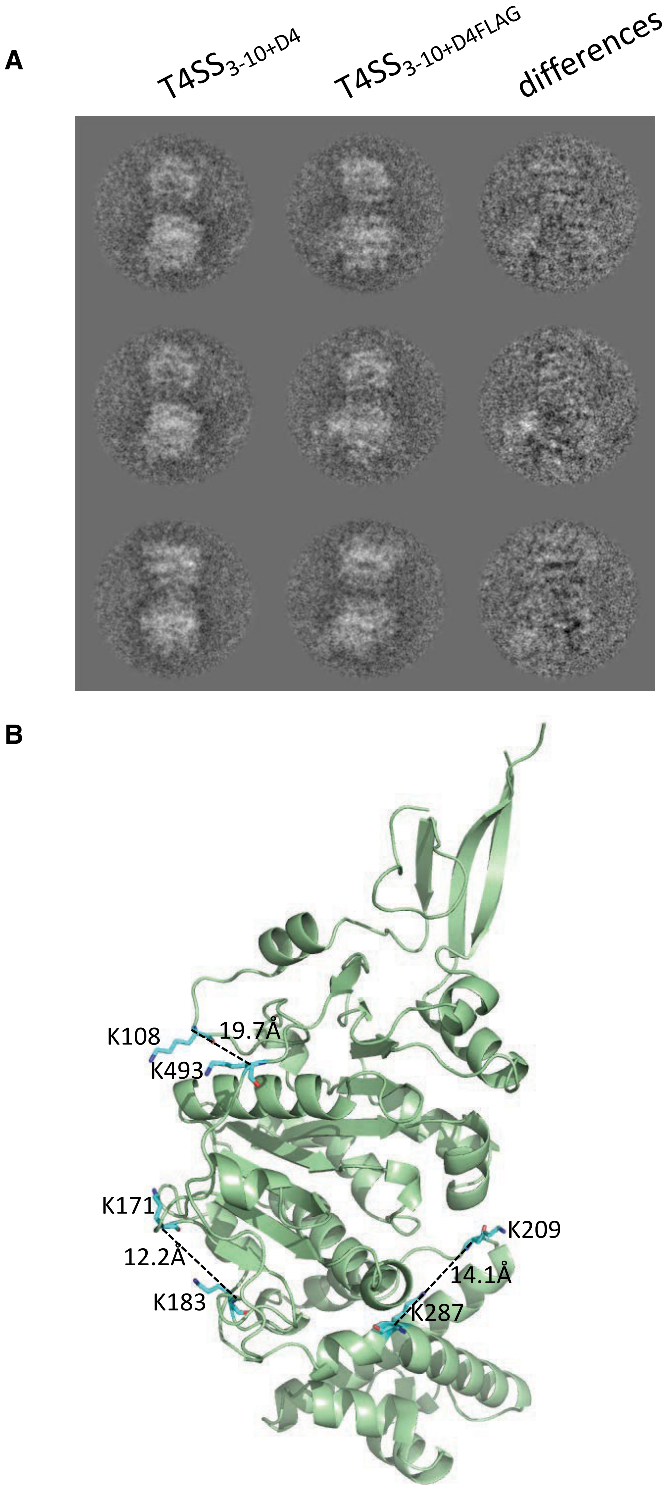

Figure EV3. Confirmation of the localization of TrwB$_{/VirD4}$.

A Difference density between class averages. Selected class averages of the T4SS$_{3\text{-}10+D4}$ complex (left column), corresponding class averages of the antibody-bound T4SS$_{3\text{-}10+D4FLAG}$ complex (middle column). The third column represents the difference between column two and column one. The whitest areas indicate the position of the bound antibody.

B Structure of TrwB$_{/VirD4}$ cytoplasmic domain. The structure is shown in ribbon representation coloured in pale green. Lysine residues involved in observed cross-links are shown as well as the distance separating them. Only one cross-link is not shown, that between residues 398 and 446 because residue 398 is in a disordered region of the structure.

© 2017 The Authors
