# Supplementary figures and images for: Structure of a VirD4 coupling protein bound to a VirB type IV secretion machinery
Source: EMBO J. 2017 Sep 18;36(20):3080–95. doi: 10.15252/embj.201796629 (PMC5916273; doi:10.15252/embj.201796629)

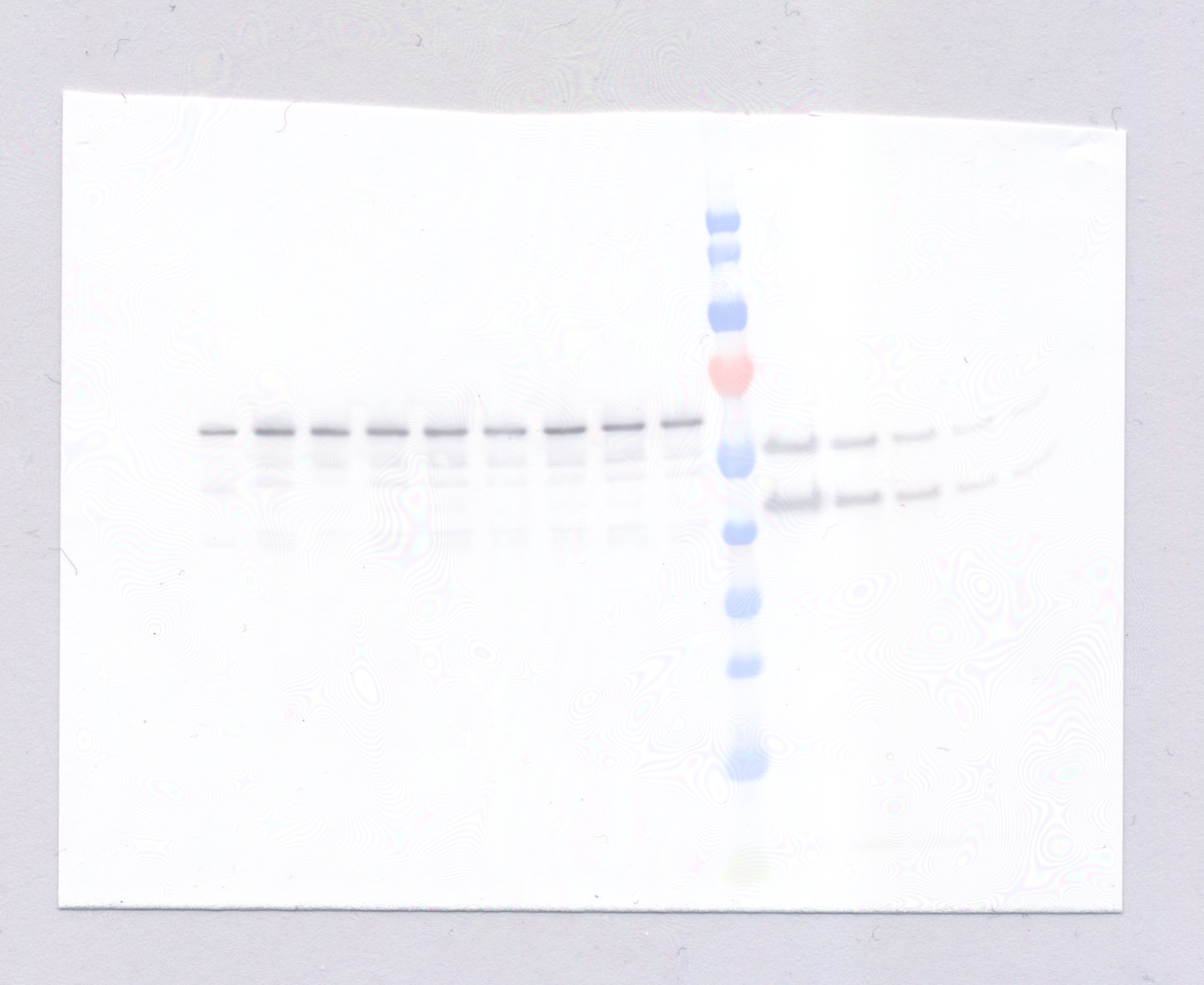

Supplement: Supplementary file 3 — Source Data for Expanded View [file EMBJ-36-3080-s006.zip › SourceFileForExpandedView/Figure_EV1_A.jpg]

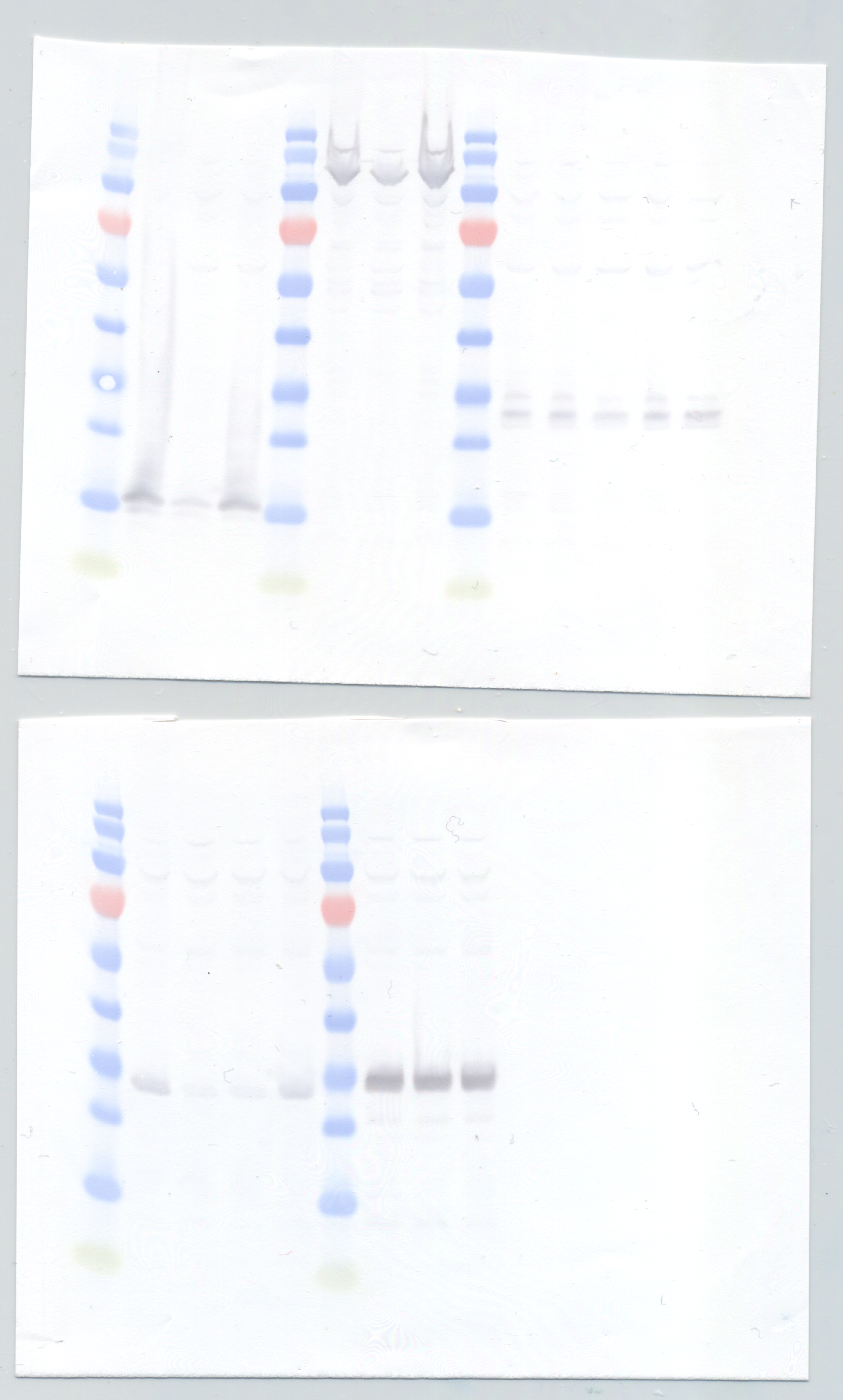

Supplement: Supplementary file 3 — Source Data for Expanded View [file EMBJ-36-3080-s006.zip › SourceFileForExpandedView/Figure_EV1_B.tiff]

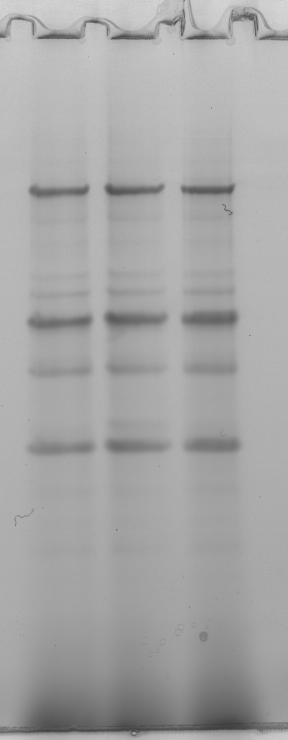

Supplement: Supplementary file 5 — Source Data for Figure 1 [file EMBJ-36-3080-s003.zip › SourceDataForFigure1/Figure_1B.tif]

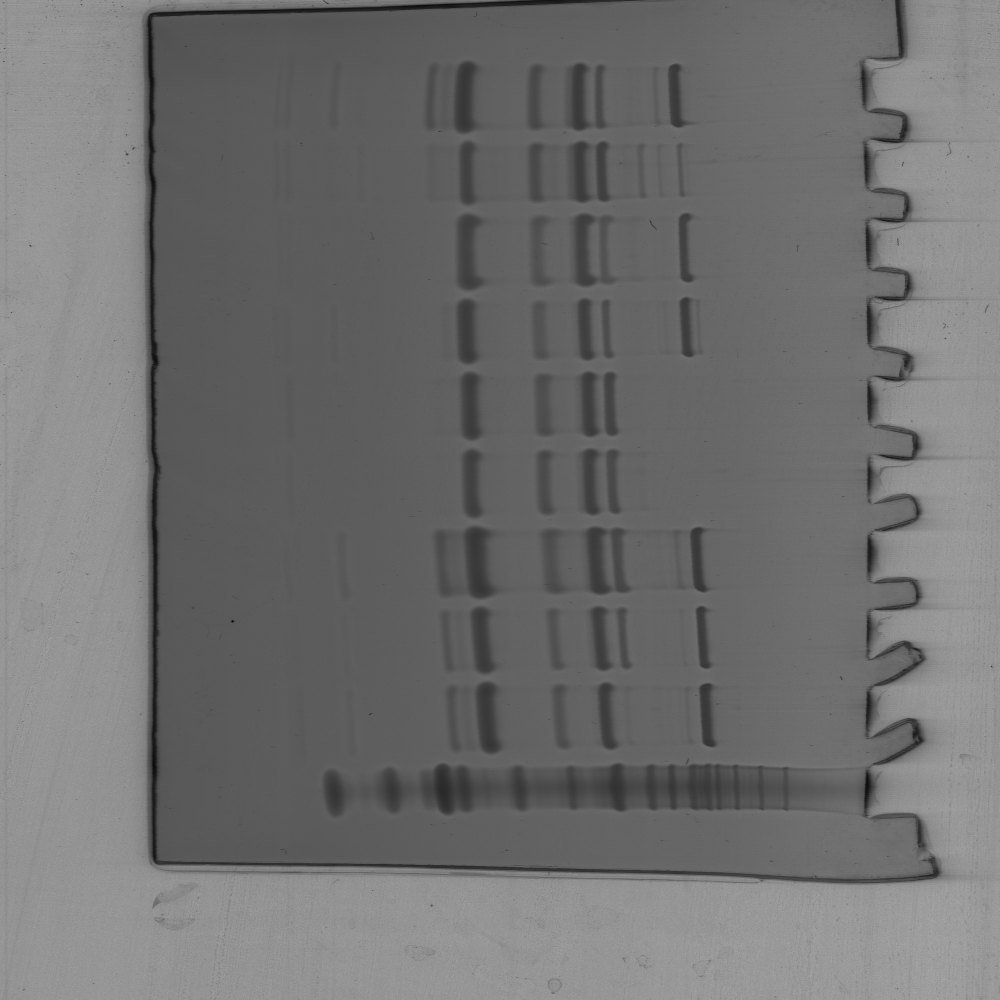

Supplement: Supplementary file 5 — Source Data for Figure 1 [file EMBJ-36-3080-s003.zip › SourceDataForFigure1/Figure_1_A-C.tif]

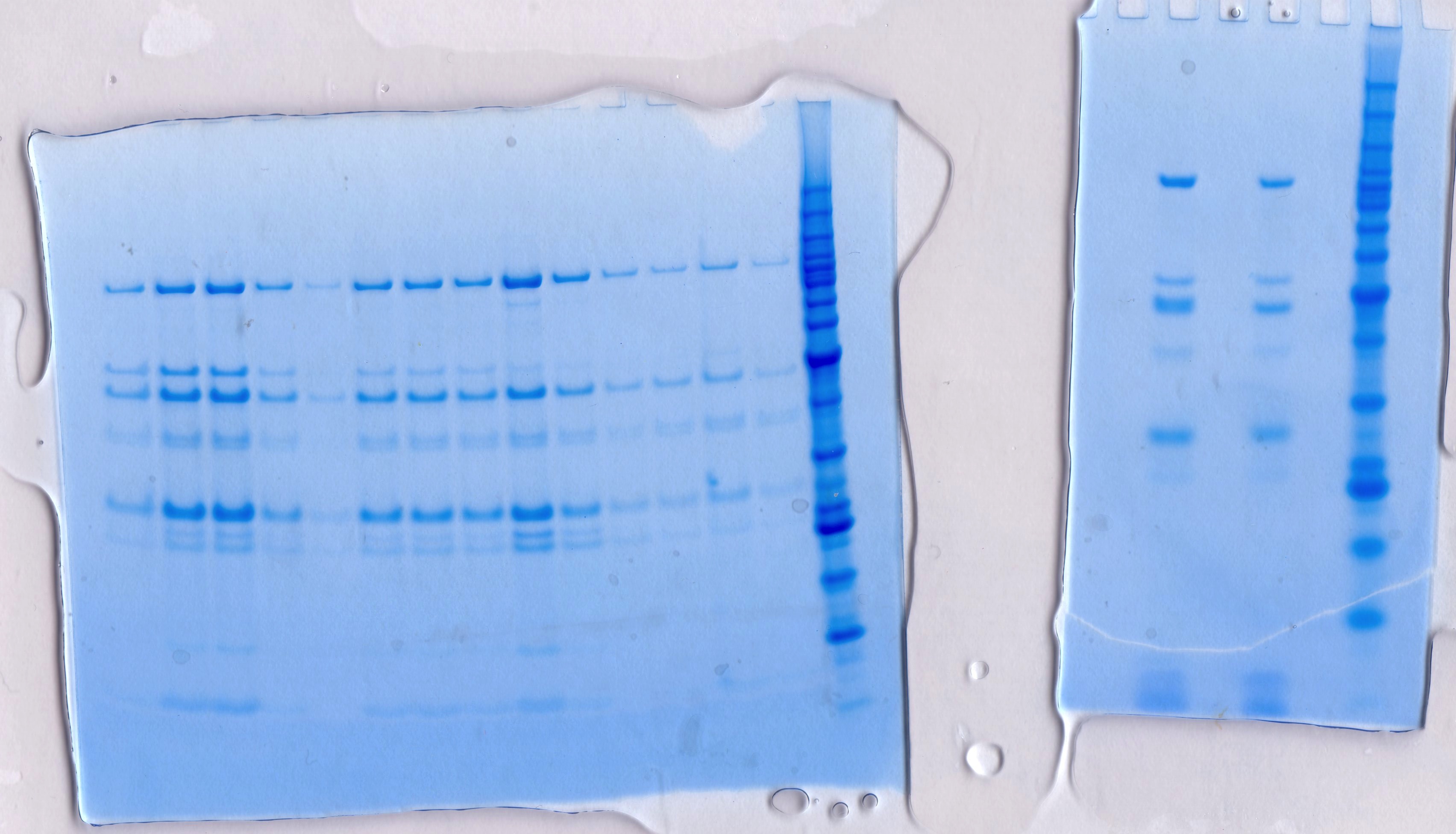

Supplement: Supplementary file 5 — Source Data for Figure 1 [file EMBJ-36-3080-s003.zip › SourceDataForFigure1/Figure_1_A.jpg]

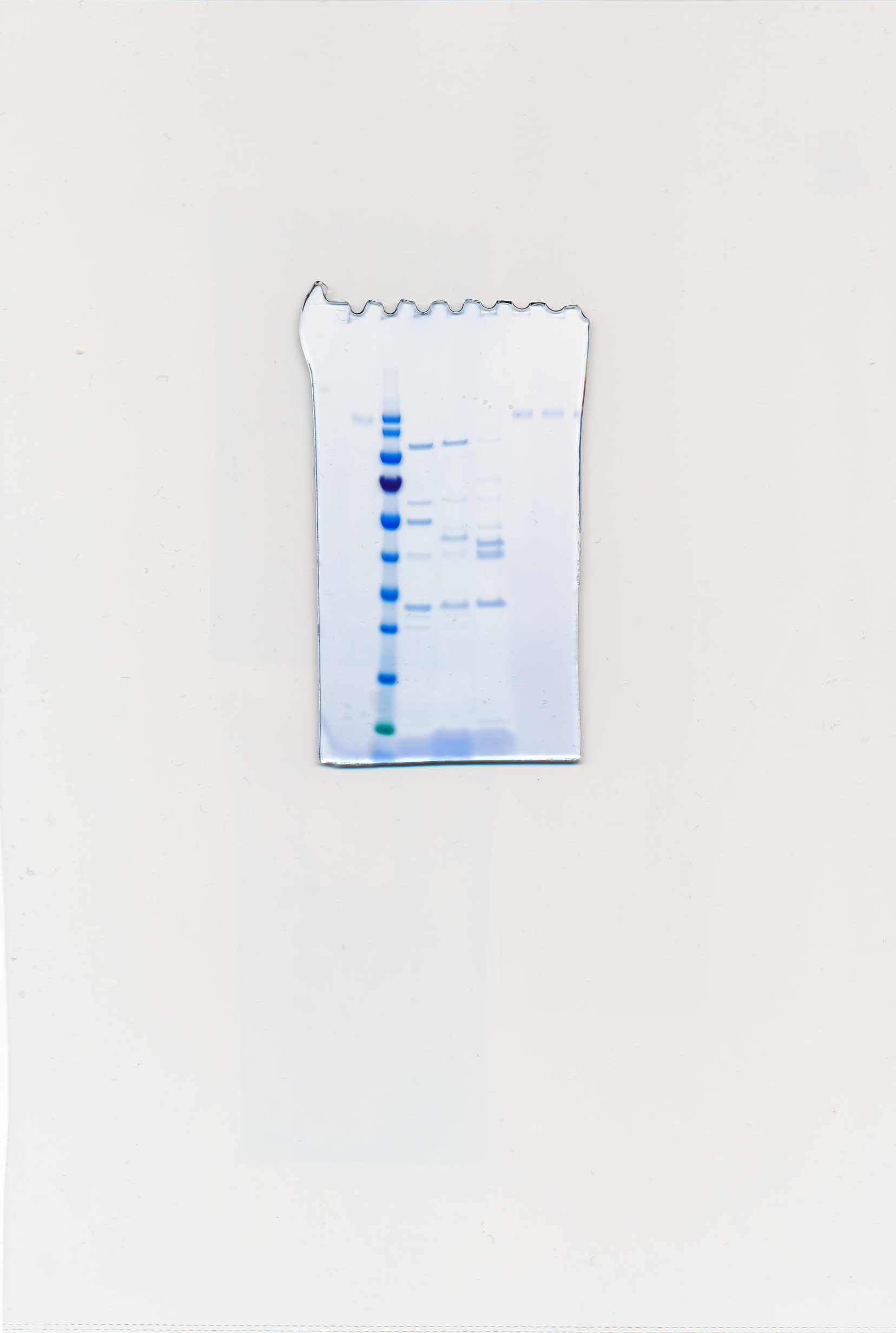

Supplement: Supplementary file 6 — Source Data for Figure 2 [file EMBJ-36-3080-s004.zip › SourceDataForFigure2/Figure_2_A.tif]

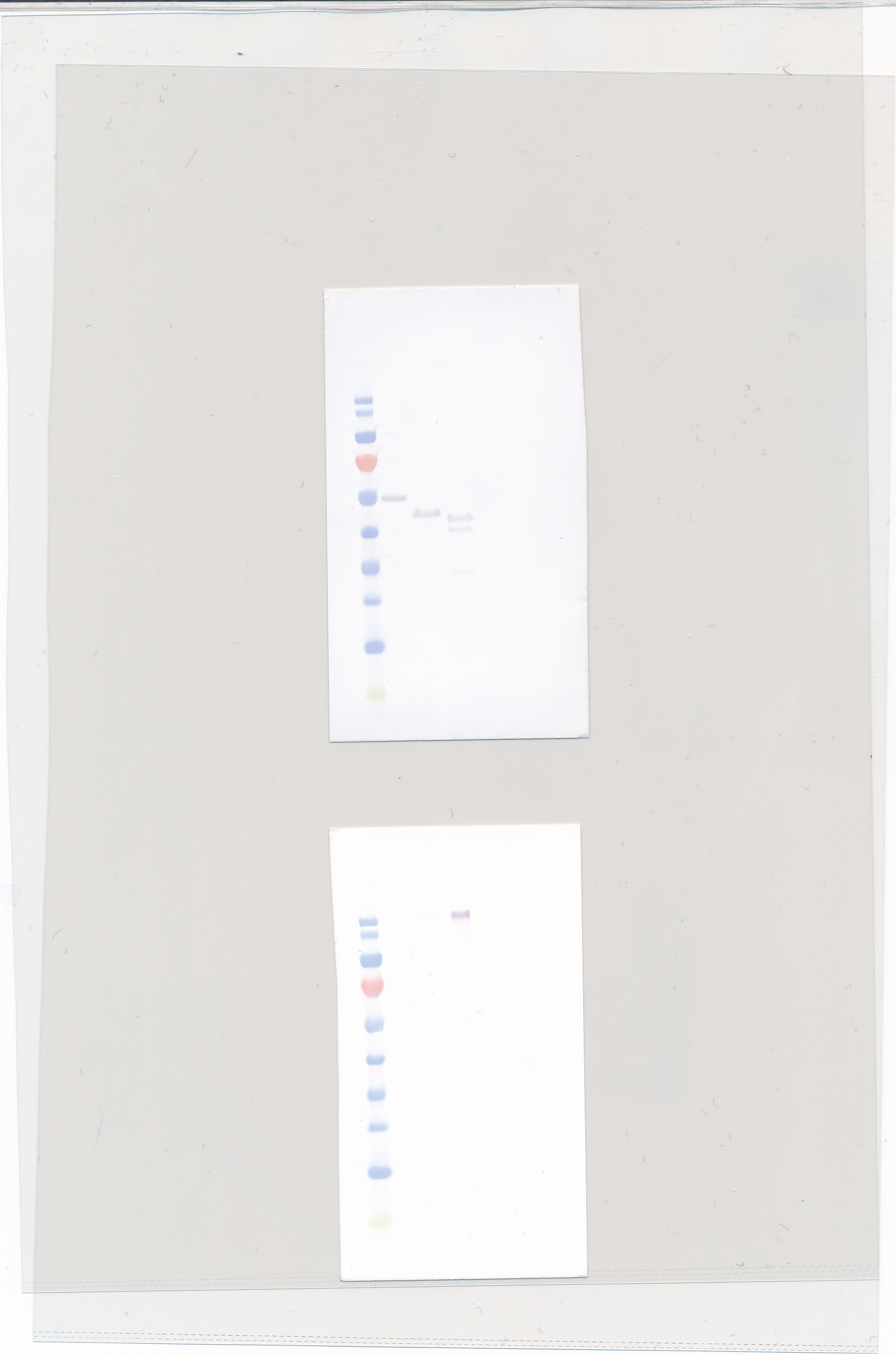

Supplement: Supplementary file 6 — Source Data for Figure 2 [file EMBJ-36-3080-s004.zip › SourceDataForFigure2/Figure_2_B.tif]

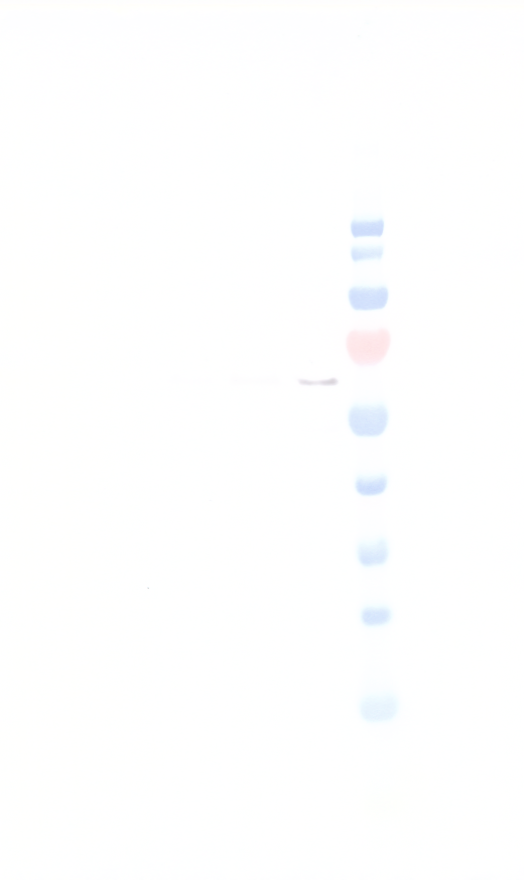

Supplement: Supplementary file 6 — Source Data for Figure 2 [file EMBJ-36-3080-s004.zip › SourceDataForFigure2/Figure_2_C.tif]

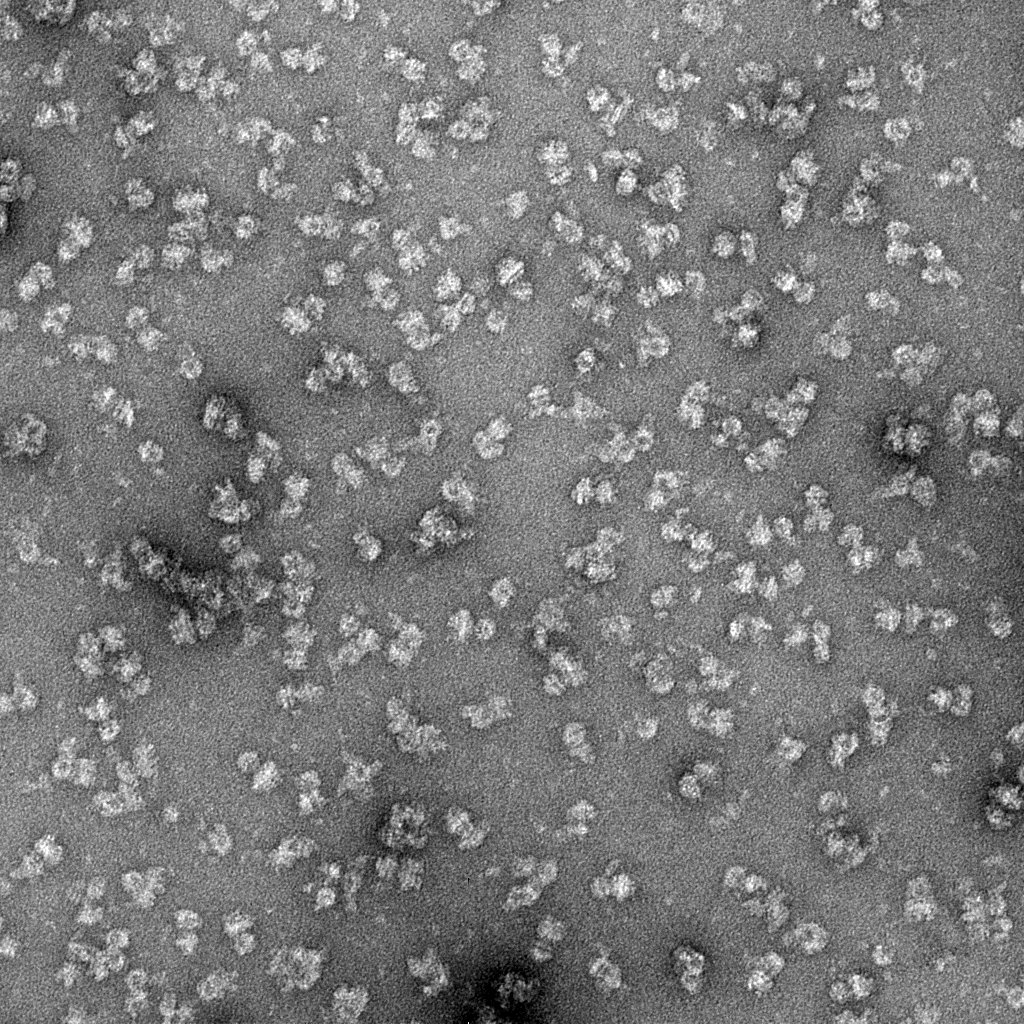

Supplement: Supplementary file 7 — Source Data for Figure 3A [file EMBJ-36-3080-s005.jpg]
